# Inflammatory Profiles in Depressed Adolescents Treated with Fluoxetine: An 8-Week Follow-up Open Study

**DOI:** 10.1155/2018/4074051

**Published:** 2018-12-18

**Authors:** Gilberto Pérez-Sánchez, Enrique Becerril-Villanueva, Rodrigo Arreola, Gabriela Martínez-Levy, María Eugenia Hernández-Gutiérrez, Marco A. Velasco-Velásquez, Samantha Alvarez-Herrera, Carlos Cruz-Fuentes, Lino Palacios, Francisco de la Peña, Lenin Pavón

**Affiliations:** ^1^Laboratory of Psychoimmunology, National Institute of Psychiatry Ramón de la Fuente, Calzada México-Xochimilco 101, Colonia San Lorenzo Huipulco, Tlalpan, 14370 México City, Mexico; ^2^Psychiatric Genetics Department, Clinical Research Branch, National Institute of Psychiatry Ramón de la Fuente, Calzada México-Xochimilco 101, Colonia San Lorenzo Huipulco, Tlalpan, 14370 México City, Mexico; ^3^Neuropharmacology Laboratory, Clinical Research Branch, National Institute of Psychiatry Ramón de la Fuente, Calzada México-Xochimilco 101, Colonia San Lorenzo Huipulco, Tlalpan, 14370 México City, Mexico; ^4^School of Medicine, National Autonomous University of México, Ciudad Universitaria, 04510 México City, Mexico; ^5^Adolescent clinic, Clinical Services, National Institute of Psychiatry Ramón de la Fuente, Calzada México-Xochimilco 101, Colonia San Lorenzo Huipulco, Tlalpan, 14370 México City, Mexico

## Abstract

Changes in cytokine levels in major depression and during treatment have been reported in adults. However, few studies have examined cytokine levels in an adolescent sample despite this being a common age of onset. *Methods*. We measured proinflammatory (IL-2, IFN-*γ*, IL-1*β*, TNF-*α*, IL-6, IL-12, and IL-15) and anti-inflammatory (IL-4, IL-5, IL-13, IL-1Ra, and IL-10) cytokine serum levels in 22 adolescents with major depression and 18 healthy volunteers. Cytokines were measured by multiplex bead-based immunoassays at baseline, and 4 and 8 weeks after commencement of fluoxetine administration in the clinical group. *Results*. Compared to healthy volunteers, adolescents with major depression at baseline showed significant increases in all pro- and anti-inflammatory cytokines, except IL-1Ra and IL-10. Significant changes were observed in fluoxetine treatment compared to baseline: proinflammatory cytokines IFN-*γ*, IL-1*β*, TNF-*α*, IL-6, IL-12, and IL-15 were decreased only at week 4 whereas IL-2 was increased only at week 8; anti-inflammatory cytokines IL-4 and IL-5 were increased at week 8 while IL-1Ra was reduced only at week 4. There were no significant correlations between cytokine levels and symptomatic improvement in HDRS. *Discussion*. The results suggest a significant interplay between cytokine levels, the depressive state, and the stage of treatment with an SSRI. To the best of our knowledge, this is the first report in depressed adolescents with elevated IL-12, IL-13, and IL-15 levels. Further studies are necessary to clarify the role and mechanisms of altered cytokine levels in the pathogenesis and physiopathology of major depressive disorder.

## 1. Introduction

MDD is a world health problem that affects people of all ages. In adolescents, MDD has increased significantly in the past ten years, with estimated prevalence rates ranging between 2 and 5.6% [1]. When occurring in children or adolescents, MDD affects their physical development as well as emotional and social conditions, which in turn produces adverse effects on their academic performance [1, 2]. Moreover, MDD is considered a relevant risk factor of suicide and places second in leading causes of death among adolescents [3]. MDD is associated with not only mood disorders but also changes in the immune response, producing a significant increment in circulatory levels of cytokines [4, 5]. Maes et al., (1992) proposed that immune response dysregulation contributes to depression symptoms [6], as demonstrated in clinical studies. It has been described that depressed patients show hyperactivity of the hypothalamic-pituitary-adrenal axis (HPA) and a subsequent increase in cortisol [7], which in turn stimulates the production of cytokines [8, 9]. Furthermore, Maes et al. reported high levels of leukocytes in depressed patients [6], and Lima and Urbina demonstrated that leukocytes express serotonergic receptors [10]. Similarly, it was shown that certain cytokines and monocytes can cross the blood-brain barrier and affect behavior [11, 12]. All these suggest that there may be a *neuro-immuno-endocrinological* interaction, and hence, whatever disturbance in this interactome could be related, directly or indirectly, to the psychiatric symptoms. Several works on adults diagnosed with MDD have described alterations in the levels of proinflammatory cytokines such as TNF-*α*, IL-1*β*, and IL-6 concerning the healthy volunteers [4, 5, 13]. Also, it has been observed that these disturbances in the cytokine levels were modified by antidepressant drugs [7, 14, 15]. The disturbances reported on the components of immune response (IR) have not been fully clarified yet; however, it has been suggested that the dysregulation of the IR is produced by a wrong or aberrant connection between the central nervous system (CNS) and the immune system [12, 16], resulting in a pathological state [17].

Currently, there are few works on children and adolescents in relation to cytokine profiles and their corresponding clinical follow-up. Some works performed in children and adolescents have reported cytokine disturbances similar to those described in adults, such as the case of IL-1*β*, IL-2, IL-10, IFN-*γ*, IL-6, and IL-8 [18–22]. Adolescents with MDD and suicidal tendencies have been reported to have significantly lower levels of TNF-*α*, as compared to those without suicidal tendencies and healthy volunteers [21]. In addition, high levels of IL-1*β* and decreased TNF-*α* were observed in children and adolescents with dysthymia [22]. It is worth noting that the levels of cytokines in adolescents usually vary with age, which affects the balance between anti-/proinflammatory components [23, 24].

This work aimed at detecting alterations in the cytokine profiles of adolescents with a first episode of MDD during eight weeks of clinical follow-up (treatment with fluoxetine) as well as the correlation between symptomatology and inflammatory profiles.

## 2. Materials and Methods

### 2.1. Subjects

The Adolescent Clinic of Instituto Nacional de Psiquiatría Ramón de la Fuente, Mexico City, assessed 337 individuals and recruited a total of 22 patients that met the inclusion criteria from January 2006 to December 2008. The inclusion criteria consisted of patients between ages 14 and 19 diagnosed with moderate MDD according to the DSM-IV-TR. The patients had a minimum baseline score in the HDRS equal or higher than 14.

All included patients agreed to participate in this study and signed the informed consent forms. Patient recruitment was done following the experimental clinical procedures of the INPRF-2035 research protocol and approved by the ethics committee of Instituto Nacional de Psiquiatría, México. Eighteen healthy volunteers (HV) aged between 14 and 19 participated in this study and were considered the reference group.

### 2.2. Clinical Procedures

Psychiatrists diagnosed all the subjects, while the clinical status of adolescents with MDD was determined using the validated Spanish version of the 21-item HDRS [25]. Patients were free of any antidepressant treatment for at least three weeks before starting the study. After a detailed explanation of the study aims, the subjects signed the written informed consent. All patients were given an SSRI (fluoxetine). During the screening visit, after performing the HDRS, every subject underwent laboratory tests to rule out any medical illnesses.

**Figure 1:**
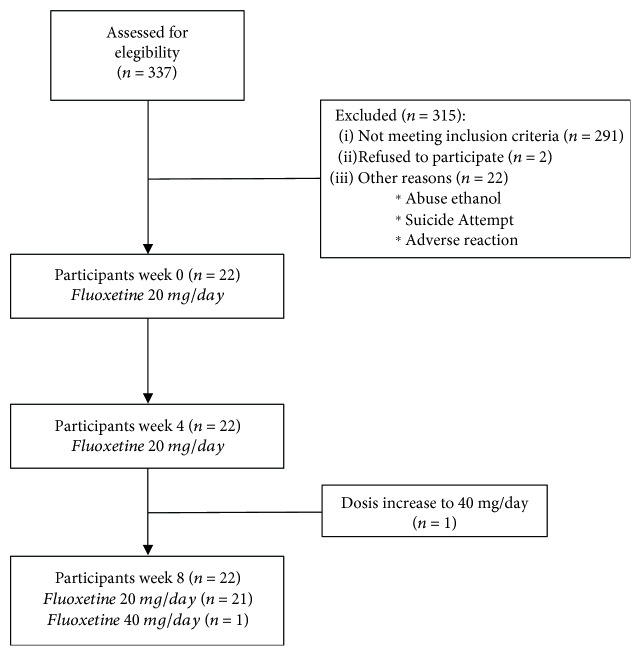
Flow diagram of eight-week fluoxetine treatment in adolescents with major depressive disorder.

Throughout the study, all patients were evaluated monthly by their psychiatrist, who applied the HDRS. Blood samples were taken from patients at baseline (week 0, W0) and weeks 4 (W4) and 8 (W8) of antidepressant treatment.  Figure 1 shows the total number of patients evaluated throughout the study as well as any changes in pharmacological treatment and reasons for withdrawal from the protocol.

### 2.3. Drugs

Fluoxetine dose was 20 mg/day. The dose was established for each patient by their psychiatrist and was adjusted when necessary throughout the study. All patients purchased their drug.

### 2.4. Serum Samples

Peripheral blood (10 mL) was collected by venipuncture from the ratio humeral venous plexus into Vacutainer® SST™ tubes with gel for serum separation (REF: 367988 BD Vacutainer System, Franklin Lakes, NJ. USA). Blood samples were centrifuged immediately (1125 × g) at 4°C for 15 minutes to obtain serum. Serum samples were separated into aliquots and stored at −80°C until analysis.

### 2.5. Cytokine Measurements

Levels of human cytokines (IL-2, IFN-*γ*, IL-1*β*, TNF-*α*, IL-6, IL-12p70, IL-15, IL-4, IL-5, IL-13, IL-1Ra, and IL-10) were measured in serum using the Bio-Plex Pro™ Human Cytokine 27-Plex Assay kit (Bio-Rad Laboratories Inc., CA, USA; cat. #M500KCAF0Y, lot. #5029511) according to the manufacturer's instructions. Analytes were detected by the addition of streptavidin-phycoerythrin and quantified in a Bio-Plex MAGPIX™ Multiplex Reader (Bio-Rad Laboratories Inc., CA, USA). Analyte concentrations were calculated by interpolation using standard curves with Bio-Plex Manager™ Software (Bio-Rad Laboratories Inc., CA, USA; version 6.1).

### 2.6. Statistical Analysis

Statistical analysis of HDRS results was performed by one-way ANOVA for repeated measures with Bonferroni's *post hoc*. Statistical differences in cytokine levels between adolescents with MDD at baseline (W0) and healthy volunteers (HV) were evaluated by Mann–Whitney *U*–Test. Statistical differences in cytokine levels along the clinical follow-up (W0 vs. W4, W8) were evaluated by one-way ANOVA for repeated measures using Dunnett's *post hoc*. The baseline (W0) was considered as a control for Dunnett's posttest. Spearman's rank correlation was used to evaluate the correlation between HDRS scores and cytokine levels along eight weeks. Statistical analysis of demographic data was performed by Student's *T*-test. Statistical analyses were performed using SigmaStat 3.5 (Systat Software, Inc.). The Z-score of Mann–Whitney *U*–Test was calculated using Social Science Statistics Tools (http://www.socscistatistics.com).

## 3. Results

### 3.1. Participants and Demographic Data

Twenty-two adolescents with MDD who met the inclusion criteria participated in this study. The demographic characteristics of the patients group as well as the reference group with 18 healthy volunteers are described in Table 1. A clinical follow-up was performed for eight weeks, considering the sampling times as follows: baseline (W0), four weeks of fluoxetine consumption (W4), and eight weeks of fluoxetine consumption (W8).

### 3.2. HDRS

**Figure 2:**
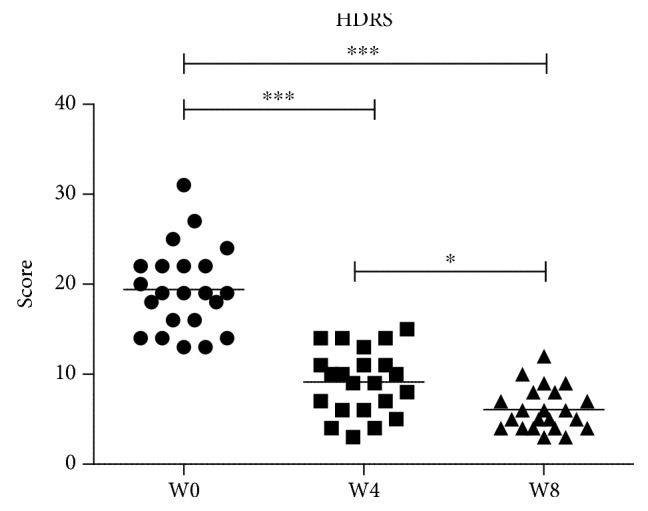
Clinical score of HDRS test in adolescents with MDD during eight weeks of clinical follow-up with fluoxetine. W0: baseline; W4: four weeks of fluoxetine treatment; W4: eight weeks of fluoxetine treatment. Statistical analysis was performed by one-way ANOVA with Bonferroni's *post hoc*. ^∗^*P* < 0.05; ^∗∗∗^*P* < 0.001.

The results of the HDRS scale show that the adolescents had improvement in the symptomatology of MDD at four (W4) and eight weeks (W8) of treatment with fluoxetine. One-way ANOVA for repeated measures showed a significant difference between treatments with an *F* = 86.75; df = 2/65, *P* < 0.0001. The Bonferroni's multiple comparison tests are shown in Figure 2.

### 3.3. Cytokine Levels

**Figure 3:**
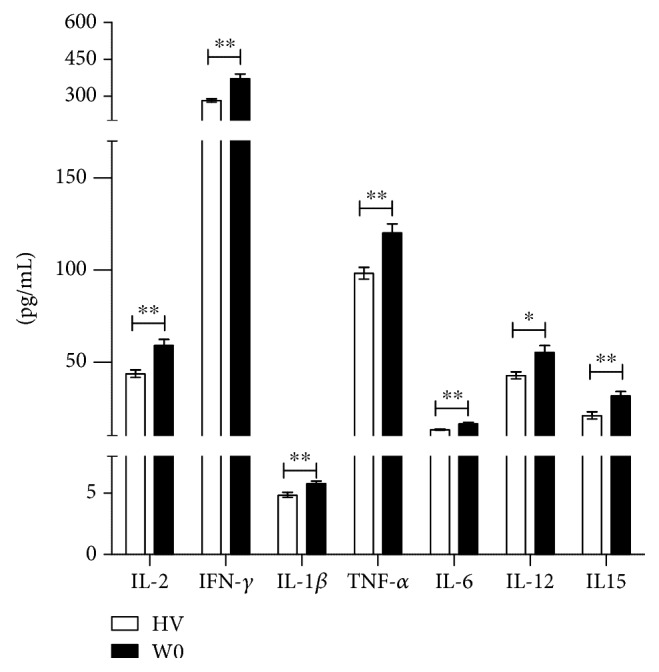
Proinflammatory cytokine serum level comparison between healthy volunteers and adolescents with MDD at baseline (previous to fluoxetine treatment). ^∗^*P* < 0.05; ^∗∗^*P* < 0.01. Mann–Whitney rank sum test was performed for statistical analysis.

**Figure 4:**
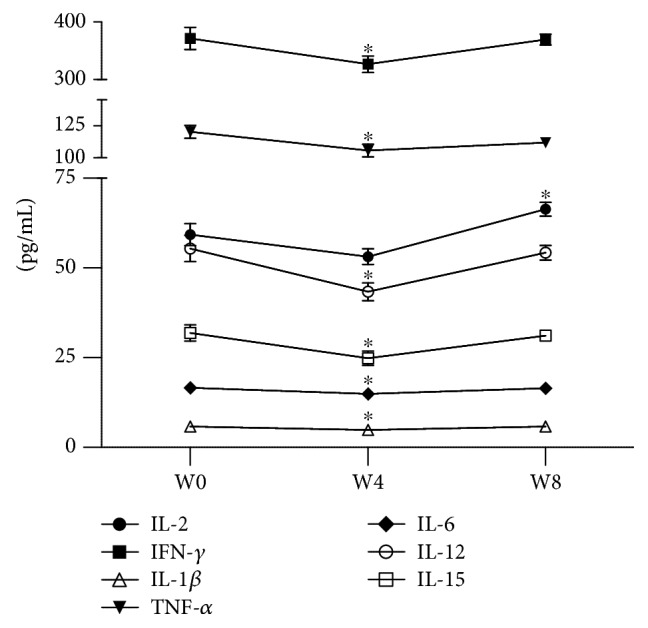
Proinflammatory cytokine serum levels of adolescents with MDD during eight weeks of treatment with fluoxetine. W0: baseline; W4: four weeks of fluoxetine treatment; W8: eight weeks of fluoxetine treatment. Statistical analysis was performed by one-way ANOVA for repeated measures using Dunnett's *post hoc*. ^∗^*P* < 0.05.

**Figure 5:**
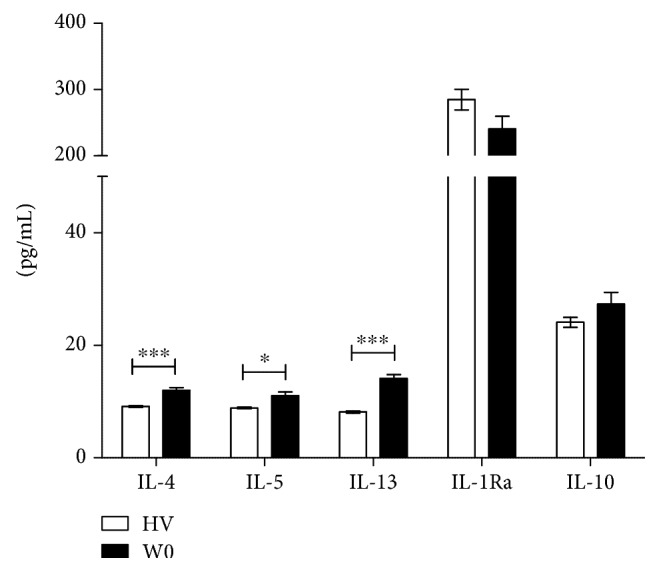
Anti-inflammatory cytokine serum level comparison between healthy volunteers and adolescents with MDD at baseline (without fluoxetine treatment). ^∗^*P* < 0.05; ^∗∗∗^*P* < 0.001. Mann–Whitney *U*–Test was performed for statistical analysis.

**Figure 6:**
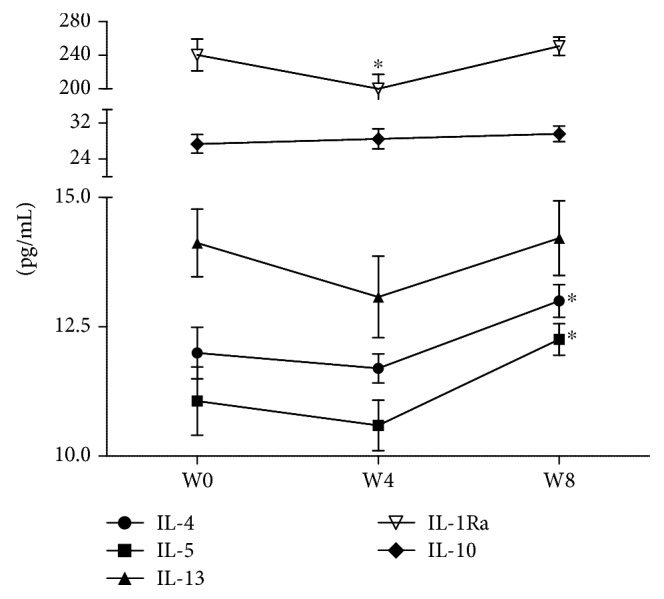
Anti-inflammatory cytokine serum levels of adolescents with MDD during eight-week treatment with fluoxetine. W0: baseline; W4: four weeks of fluoxetine treatment; W8: eight weeks of fluoxetine treatment. Statistical analysis was performed by one-way ANOVA for repeated measures using Dunnett's *post hoc*. ^∗^*P* < 0.05.

The cytokine levels in the serum of MDD adolescents were measured at baseline (W0) and weeks four (W4) and eight (W8) of treatment. The results are described below. Figures 3–6 present the mean (pg/mL) of each cytokine ± SEM (standard error of the mean).

#### 3.3.1. Proinflammatory Cytokine Levels

*(1) MDD W0 vs. HV*. The proinflammatory cytokines showed an increase in MDD adolescents at baseline as compared to the healthy volunteers (HV). The Mann–Whitney *U*–Test showed significant differences for IL-2 (*Z* = 3.08565; *P* < 0.01), IFN-*γ* (*Z* = 2.96331; *P* < 0.01), IL-1*β* (*Z* = 2.8409; *P* < 0.01), TNF-*α* (*Z* = 3.23518; *P* < 0.01), IL-6 (*Z* = 2.86816; *P* < 0.01), IL-12p70 (*Z* = 2.16132; *P* < 0.05), and IL-15 (*Z* = 2.94972; *P* < 0.01), (Figure 3).

*(2) Clinical Follow-up*. The one-way ANOVA for repeated measures showed a significant difference between treatments for IL-2 (*F* = 11.943; *P* < 0.0001), IFN-*γ* (*F* = 7.935; *P* < 0.01), IL-1*β* (*F* = 15.58; *P* < 0.0001), TNF-*α* (*F* = 5.074; *P* < 0.05), IL-6 (*F* = 3.354; *P* < 0.05), IL-12p70 (*F* = 11.74; *P* < 0.05), and IL-15 (*F* = 7.872; *P* < 0.01); in all cases, df = 2/65.

Dunnett's multiple comparisons showed that IL-2 significantly increased (*P* < 0.05) at week eight (W8) of treatment with fluoxetine compared to the levels of the cytokine at baseline (W0). In contrast, the other six proinflammatory cytokines showed a significant decrease at four weeks of treatment (W4): IFN-*γ* (*P* < 0.05), IL-1*β* (*P* < 0.05), TNF-*α* (*P* < 0.05), IL-6 (*P* < 0.05), IL-12p70 (*P* < 0.05), and IL-15 (*P* < 0.05), but their levels were restored at week eight (W8) (Figure 4).

#### 3.3.2. Anti-Inflammatory Cytokine Levels

*(1) MDD W0 vs. HV*. Anti-inflammatory cytokines IL-4, IL-5, and IL-13 were significantly higher in MDD adolescents at baseline (W0), as compared to healthy volunteers. The Mann–Whitney *U*–Test showed significant differences for IL-4 (*Z* = 3.76531; *P* < 0.001), IL-5 (*Z* = 2.48755; *P* < 0.05), and IL-13 (*Z* = 4.9887; *P* < 0.001), whereas no changes were found in the levels of IL-1Ra and IL-10 (Figure 5).

*(2) Clinical Follow-up*. The one-way ANOVA for repeated measures showed a significant difference between treatments for IL-4 (*F* = 8.334; *P* < 0.001), IL-5 (*F* = 6.764; *P* < 0.01), and IL-1Ra (*F* = 4.943; *P* < 0.05); in all cases, df = 2/65.

Dunnett's multiple comparisons showed that IL-1Ra levels significantly decreased (*P* < 0.05) after four weeks (W4) of treatment but were restored four weeks later (W8). In contrast, IL-4 and IL-5 were significantly increased (*P* < 0.05) at the endpoint (W8) of the clinical follow-up. The levels of IL-13 and IL-10 did not change throughout the clinical follow-up (Figure 6).

## 4. Discussion

MDD has been little studied in adolescents; therefore, there are scarce reports on cytokine profiles along drug treatment. Most of the works on MDD are *cross-sectional* studies because of the low adherence of patients to their treatment, which makes it difficult to perform a clinical follow-up. There are reports in both lymphocyte subsets [15] and cytokine profiles in MDD patients along treatment with SSRIs until 52 weeks of clinical follow-up [26]; however, such studies have been carried out in adults. Other works have reported longitudinal studies in children and/or adolescents; nonetheless, they were limited to measuring no more than three inflammatory markers [27].

In this work, we present the proinflammatory (IL-2, IFN-*γ*, IL-1*β*, TNF-*α*, IL-6, and IL-12) and the anti-inflammatory (IL-15, IL-4, IL-5, IL-13, IL-1Ra, and IL-10) serum cytokine profiles of MDD adolescents. All these cytokines were measured at baseline (W0) and four (W4) and eight weeks (W8) of fluoxetine treatment.

### 4.1. Proinflammatory Cytokines

#### 4.1.1. IL-2

Several clinical studies have reported disturbances in the levels of IL-2 in either adult or adolescent MDD patients. In this study, we observed that IL-2 was significantly higher in MDD adolescents than in healthy volunteers, which is in agreement with previous reports in adolescents with mood disorders [19, 28]. In contrast, the studies concerning adult patients are controversial since there are reports of both high [29–32] and low values [7, 26, 33, 34]. The effects of SSRIs on IL-2 levels depend on the drug being used; while sertraline does not affect IL-2 levels in adult patients [30], fluoxetine lowers them after 20 weeks of administration [34]. However, our results in adolescents showed a significant rise of IL-2 at week eight of fluoxetine administration. Another interesting issue reported in MDD adults administered with SSRIs (fluoxetine, paroxetine, sertraline, and escitalopram) was the effects upon the subpopulations of lymphocytes, such as fluctuations of T helper cells along the clinical follow-up [15]. These fluctuations in T helper cells could be related to the inconsistencies in the disturbances of IL-2 that have been reported. In addition, immunotherapy with IL-2 can produce depressive symptoms, which in turn are reduced by the administration of antidepressants [35–37], supporting the role of IL-2 in depressive disorders. Although we know IL-2 therapy induces depressive disorder, little is known on the molecular mechanisms exerted by IL-2 to trigger such disorder. However, it is well-known that IL-2 is also expressed in the central nervous system (CNS), where it acts modulating the release of serotonin, dopamine, and acetylcholine. This modulation of neurotransmitters depends on the concentration of IL-2; while low concentrations reduce the release of neurotransmitters, high concentrations suppress it [38]. Interestingly, studies in murine models have demonstrated that chronic rise in cytokine levels, such as IL-2, may increase the permeability of the blood-brain barrier (BBB) and allow IL-2 to reach the brain [38, 39]. The above could partially explain the likely effects that IL-2 may be exerting on adolescents with MDD.

#### 4.1.2. IFN-*γ*

Just like IL-2, disturbances in IFN-γ levels have been reported in MDD patients. In our work, IFN-γ was significantly higher in MDD adolescents, as previously reported [20, 21], yet no differences have been found between suicidal and nonsuicidal patients [21]. Moreover, it is reported that antidepressants do not affect the levels of IFN-*γ* in MDD adolescents [19, 20, 28], which is partly in agreement with our results because we observed only temporal changes at W4. In contrast, studies in adults report lower levels of IFN-*γ* in MDD patients compared to controls [7, 26, 33, 34]. Pavón et al. found significantly lower levels of Th1 cytokines (IFN-*γ* and IL-2) in MDD patients without treatment, and these changes were attributable to high levels of cortisol [7] as previously discussed. Marquesdeak et al. analyzed the cytokine profiles in women with different subtypes of MDD, but they did not find disturbances in IFN-*γ* levels. However, they demonstrated that treatment with imipramine or sertraline increases IFN-*γ* concentrations [40]. The work of Hernández et al. also described that the treatment with SSRIs increased IFN-*γ* levels after 52 weeks of follow-up, which correlated with a decrease in cortisol, suggesting a partial reestablishment of the HPA axis function due to the long-term treatment with SSRIs [34]. Then, it is likely that the effects of medication on IFN-*γ* levels can be detected in a clinical follow-up longer than eight weeks.

IFN-*γ*-induced depression has been less studied than that of depression induced by IFN-*α*, whose mechanisms are well documented [41–47]. However, there are some reports of cases in which patients receiving IFN-*γ* therapy have exhibited mood disorders and depression [42]. A study in patients with hepatitis C treated with IFN-*α* demonstrated that carriers of IFNG (+874) T allele are more likely to present IFN-*α*-induced depression [48]. It should be noted that both IFN-*α* and IFN-*γ* upregulate IDO (indoleamine-2,3-dioxygenase) enzyme, which in turn favors the synthesis of kynurenines instead of serotonin, triggering depression [39, 45, 49, 50]. Moreover, IFN-*γ* can induce the generation of reactive oxygen species (ROS), which reduce the levels of BH4 (tetrahydrobiopterin). Then, the reduction of BH4 decreases the synthesis of catecholamine, affecting the neurotransmission in patients with MDD [50]. This suggests that, directly or indirectly, IFN-*γ* is involved in the onset and development of depression and its high levels found in MDD adolescents are strongly related to the disorder.

#### 4.1.3. IL-1*β*

Significantly high levels of IL-1*β* have been reported in female adolescents with emotional disorders. This rise was also detected when comparing treated and nontreated SSRI subgroups, being significantly higher in the first one [19]. In children/adolescents with MDD or dysthymia and anxiety, IL-1*β* concentration was not significantly different from that of controls, and medication did not affect IL-1*β* levels [20, 28]. Nonetheless, a positive correlation was found between concentrations of IL-1*β* and scores for MDD and anxiety [22]. Our results showed significantly higher levels of IL-1*β* in MDD adolescents compared with the controls, as reported by Henje Blom et al. and Pandey et al. Additionally, we observed that four weeks after fluoxetine treatment (W4), IL-1*β* values were significantly reduced, but they increased again at W8. In MDD adults, reports on IL-1*β* describe significantly lower levels of IL-1*β* in MDD patients without treatment, regarding the control group [34], yet these values are increased after 20 weeks of SSRI treatment, being significantly higher than those of the controls at week 52 [26]. Interestingly, in the study mentioned above, cortisol levels were significantly higher in MDD patients at the start of SSRI treatment but decreased by 30% at week 52, which corresponded with the trend of IL-1*β* values. The work of Marquesdeak et al. also demonstrated that SSRI administration increases IL-1*β* levels [40].

Concerning our work, it is likely that extended clinical follow-up times might be useful to detect a clearer effect of fluoxetine on concentrations of IL-1*β*. On the other hand, studies in animal models have revealed that IL-1*β* acts as a potent stimulant of the serotonin transporter mediated by p38 MAPK, increasing the uptake of serotonin, which is in accordance with the physiopathology of depression. Moreover, IL-1*β* reduces neurogenesis in the hippocampus—another well-characterized event that occurs in depression [51, 52]. In fact, high levels of IL-1*β* in the hippocampus and prefrontal cortex have been evidenced by some stress-induced depression experiments in rodent models [51, 53, 54]. There is evidence that IL-1*β*-related inflammation in CNS mediated by NLPR3 inflammasome and P2X7 is strongly related to depression [45, 52, 53]. IL-1*β* has also been reported to act as an inhibitor of NMDAR-mediated long-term potentiation (LTP) [45], an essential event in the synaptic plasticity, affecting the neurotransmission in consequence. Another important issue to emphasize is the synergistic effect that IL-1*β* exerts upon IFN-*α*-induced IDO upregulation [55], an event related to depression development. These backgrounds support the role of IL-1*β* in the physiopathology of depression and give meaning to the disturbances that we found in this work.

#### 4.1.4. TNF-*α*

In our work, we found a significant increase in TNF-*α* among MDD patients at W0, regarding controls. This is in agreement with a previous work in adolescent suicide victims in which TNF-*α* was significantly elevated in the prefrontal cortex [56]. Unexpectedly, the TNF-*α* protein was significantly lower in the plasma of adolescent suicidal-attempters, regarding that of nonsuicidal subjects [21]. In contrast, other works in adolescents did not find differences between MDD patients and controls [19, 20, 22, 28]. In adults, previous reports are in accordance with our results that show a significant rise in circulatory levels of TNF-*α* in MDD patients [33, 57]. The treatment with sertraline has shown to be effective in suppressing TNF-*α* production after eight weeks of administration [30], but no significant differences were detected when different antidepressants were used and analyzed [33]. In line with the work of Kim et al. (2007), we proved a significant reduction of TNF-*α* after four weeks (W4) of fluoxetine treatment, which was restored four weeks later (W8). Another work supporting our results was presented by Amitai et al. who demonstrated a significant reduction of TNF-*α* after eight weeks of treatment with fluoxetine in children or adolescents with depression and/or anxiety disorders [58].

Several clinical trials using monoclonal anti-TNF-*α* antibodies (etanercept and infliximab), which block the inflammatory activity of TNF-*α*, have shown the influence of TNF-*α* in depression. These biodrugs have demonstrated to reduce fatigue in patients with advanced cancer and also improve depressive symptoms in antidepressant-resistant patients [45]. In the future, such medications could be used as antidepressants, with even fewer side effects than conventional drugs. Additionally, TNF-*α* also has the capability to enhance the IFN-*γ*-induced IDO expression, which in turn favors the kynurenine pathway instead of serotonin synthesis, a mechanism potentially associated with depressive symptoms [39, 45, 55]. This evidence supports the direct or indirect participation of TNF-*α* in the development of depression, which gives meaning to our finding of high levels of TNF-*α* in MDD adolescents.

#### 4.1.5. IL-6

In children and adolescents, IL-6 has been identified as a good biomarker of psychopathology. The levels of IL-6 are significantly higher in patients with a diagnosis of affective, anxiety, adjustment, psychotic, obsessive-compulsive, and Tourette disorders [18] as well as depressed patients [19, 28, 56]. We found that IL-6 was significantly increased in MDD adolescents regarding healthy volunteers. Moreover, a significant decrease after four weeks of fluoxetine treatment (W4) was detected; it should be noted that at week eight (W8) the values of IL-6 were restored. Our results are in agreement with several reports in both adolescent and adult MDD patients exhibiting high values of IL-6. In MDD adolescents, it has been demonstrated that high levels of IL-6 have a gender factor, being higher in women, but no changes due to medication was detected [28]. However, a study performed in women demonstrated that SSRI treatment can lower levels of IL-6 even lower than those of controls and the non-SSRI group [19]. Both protein and mRNA of IL-6 have been reported to be significantly higher in the prefrontal cortex of teenage suicide victims than in that of controls [56]. However, when suicidal and nonsuicidal IL-6 values were compared in plasma samples, no difference was found [21]. Reports on IL-6 in MDD adults are in agreement with our results, showing a significant increase in serum levels of MDD patients [14, 31, 57, 59]. In contrast with our results, Kim et al. demonstrated a significant decrease in IL-6 among MDD adults after six weeks of treatment with several antidepressants [33]. In addition, MDD patients who fail to respond to SSRI treatment also fail to respond to IL-6 suppression by the drug [57].

Therapy with tocilizumab, a monoclonal antibody against the IL-6 receptor, showed improvement in fatigue symptomatology among patients with rheumatoid arthritis, a disorder strongly related to depression, which positions IL-6 as a therapeutic target of depression [60, 61]. As IL-1*β*, IFN-*α*/*γ*, TNF-*α*, and IL-6 have also been related to the onset and development of depression. The polymorphism rs1800795 in the IL-6 promoter gene is a genotype that overproduces IL-6, which has been associated with depressive disorder [62]. Moreover, p38 MAPK-mediated IL-6 increases the expression of serotonin transporter and enhances its activity [45]. This results in a lower concentration of serotonin in the synaptic cleft, which is a common feature in depression. IL-6 also induces the upregulation of IDO, increasing kynurenine and reducing serotonin [39, 45]. This context provides evidence of how high concentrations of IL-6 may contribute to the onset and development of depression.

#### 4.1.6. IL-12 (p70)

The levels of IL-12p70 that we determined in this work were significantly higher in MDD adolescents regarding the control group. To date, we are not aware of other studies evaluating IL-12p70 in MDD adolescents, but the reports in adults correspond with our results, showing significantly higher levels of IL-12 in MDD patients, as compared with the control group [30, 31]. Regarding the clinical follow-up, a significant reduction of IL-12p70 was evident at the midpoint (W4) of fluoxetine treatment but not at the endpoint (W8), when the values were restored. Contrastingly, other reports have shown that sertraline lowers the levels of proinflammatory cytokines, such as IL-2 and TNF-*α*, but not IL-12 that was maintained significantly higher in patients with MDD throughout the treatment [30]. Even though they belong to the same family (SSRIs), fluoxetine and sertraline seemingly affect IL-12 in a different manner. Additionally, because no mechanisms on the participation of IL-12 in depression have been described, future studies may focus on this point.

#### 4.1.7. IL-15

Little is known about the role of IL-15 in MDD; to the best of our knowledge, this is the first report in adolescents. In adults, Simon et al. reported increased IL-15 in MDD patients [31], which is in agreement with our results. Even though fluoxetine decreased the concentrations of IL-15 after four weeks (W4) of treatment, this effect was reverted at week eight (W8).

Studies in rodents have shown that both IL-15 and IL-15 receptor (IL-15R*α*) are expressed in CNS, where they regulate not only neural stem cell proliferation but also neural stem cell differentiation, being an essential element for neurogenesis [63, 64]. Moreover, IL-15R*α* plays a key role in maintaining hippocampal GABA transmission and preventing memory deficit in mice [65]. Also, IL-15 has been reported to decrease the synaptosomal uptake of serotonin and modulate SERT [66]. This evidence reveals the positive role of IL-15 in neuroplasticity and neurogenesis. It seems that IL-15 may have a beneficial effect on psychiatric disorders instead of a deleterious one. Yet, the disturbance that we found in IL-15 and its involvement in depression comprise a very important issue to clarify in further studies.

### 4.2. Anti-Inflammatory Cytokines

#### 4.2.1. IL-4

The role of IL-4 in depression is not yet well established. Most of the research on IL-4 in MDD provides reports on adults, with a small number on adolescents. In our work, we found increased levels of IL-4 in MDD adolescents, which contradicts previous reports on patients with psychopathology [18], MDD [20], or suicide attempts [21]. In adults, the results are more similar to ours, showing higher levels of IL-4 in MDD patients as compared with the controls [7, 26, 31, 34]. However, Kim et al. and Sutcigil et al. report the opposite [30, 33]. Regarding the effects of antidepressants on cytokines, our results are in agreement with the study of Sutcigil et al., which reported a significant increase in IL-4 after eight weeks (W8) of sertraline treatment [30]. The elevation of IL-4 in MDD adolescents we found could be in response to the high levels of proinflammatory cytokines observed in the same group in order to balance the inflammatory response. Moreover, contrary to most of the proinflammatory cytokines analyzed in this work (except IL-15), which seem to have promoting effects towards depression, IL-4 appears to have beneficial effects upon psychiatric disorders in addition to the obvious anti-inflammatory effects. Experiments in animal models have shown the astrocytic production of BDNF (brain-derived neurotrophic factor) as well as beneficial effects on cognition mediated by IL-4 [67]. In addition, IL-4 is able to stimulate neural stem/progenitor cell (NSPC) proliferation and neurogenesis through STAT6 phosphorylation [68]. It is well known that BDNF is strongly related to depression. In fact, reduced levels of BDNF have been reported in depressed patients [69–72]. This suggests that high levels of IL-4 in MDD adolescents may play a dual role: the maintenance of inflammatory homeostasis and the upkeep of neurotrophic equilibrium.

#### 4.2.2. IL-5

The role of IL-5 in MDD adolescents has been scarcely studied. In 2015, Gariup et al. (2015) performed a study in children and adolescents with acute psychopathology in which a set of twelve cytokines, including IL-5, was evaluated. However, they did not find significant differences between IL-5 in patients and controls. In this work, we found elevated levels of IL-5 in MDD adolescents, as compared to healthy volunteers, which correspond to previous works in adults [73, 74]. Moreover, high levels of IL-5 have been associated with the likelihood of suffering major depression [75]. It has been seen that antidepressant-responders have higher levels of IL-5 than nonresponders [76]. Up to now, we have not found works reporting the effects of antidepressants upon IL-5, but our results showed a significant increase of IL-5 at week eight (W8) with respect to those at baseline (W0), as in the case of IL-4. Because of the little information on the role of IL-5 in depression, this work in adolescents is the basis for future studies focused on clarifying its involvement in this disorder.

#### 4.2.3. IL-13

Previous studies have revealed an increase of circulatory levels of IL-13 in MDD adults when compared to healthy volunteers [7, 26, 34, 74, 77], which is consistent with our results. Up to now, we have not found other studies reporting disturbances in IL-13 in MDD adolescents and thus its role in depression remains unclear. However, a rodent model has demonstrated a negative impact of IL-13 upon neuron viability in the hippocampal CA1 region through activation of microglial NAPDH-oxidase-derived oxidative stress [78]. Moreover, IL-13 is able to induce death in cells of microglia, which is responsible for the neuroinflammation in CNS [79, 80]. This background is relevant because neuronal and microglial degeneration may contribute not only to the development of neurodegenerative diseases but also to psychiatric disorders [81].

#### 4.2.4. IL-1Ra

The role of IL-1Ra in the development of depression has been evidenced in a model of depression in mice, in which it has been demonstrated that the IL-1/IL-1Ra signaling induces depressive-like symptoms by two potential mechanisms: the reduction of adrenocortical activation and the reduction of hippocampal neurogenesis [51, 82]. In addition, IL-1Ra prevents the reduction of BDNF in hippocampus, suggesting a cooperative role between IL-1Ra and BDNF, with a positive impact on emotional processes [39]. In this work, we did not find significant differences in the levels of IL-1Ra between MDD adolescents and healthy volunteers. However, fluoxetine significantly reduces IL-1Ra after four weeks of treatment (W4), but four weeks later (W8) its levels were restored. Our results disagree with those observed in adults by Dahl et al., who reported higher levels of IL-1Ra in MDD patients; however, they also observed that treatment with SSRIs can reduce IL-1Ra levels [73]. The mechanisms by which SSRIs affect IL-1Ra are unknown yet, but they could be clarified in further studies.

#### 4.2.5. IL-10

IL-10 alterations have been associated with depressive symptoms such as fatigue, loss of appetite, and anhedonia [50]. In our results, we did not find changes in the levels of IL-10 between MDD adolescents and healthy volunteers or throughout the treatment with fluoxetine, which is consistent with the results of Pallavi et al. (2015). But in a study performed in women, MDD patients showed significantly higher levels of IL-10 [19], suggesting that the gender factor could be affecting the study of this cytokine at least in adolescents. In adults, several studies have shown more elevated levels of IL-10 in MDD patients than in controls [26, 31, 34, 73, 76], and the treatment with SSRIs is positively correlated with the reduction in IL-10 [26, 34, 73]. Because the disturbances in IL-10 among MDD patients appear to be affected by gender and age, further studies should be focused on clarifying this point.

Alterations in cytokine levels and depression are closely related, especially in adolescents. Although the direct connection is not fully clear yet, some factors have been suggested to link them. For example, genetic polymorphisms in IL-10 and IL-6 have been associated with a higher risk of suffering depression [50, 62]. Epigenetic factors, such as alterations in the chromatin structure and/or histone acetylation patterns, have been related to depression [83, 84] and could affect cytokine expression. In addition, it has been shown that adverse childhood experiences, such as sexual abuse, also increase the risk of triggering depression in adolescence [50] as well as alterations in the levels of proinflammatory cytokines [85]. Another factor is the brain-gut axis, which also plays an important role in mental disorders. Several studies have revealed that abnormal gut microbiota that is often accompanied by abnormal expression of inflammatory cytokines [86] may cause depression. Depressed patients often have gut dysfunction, such as appetite and metabolic disturbances and other gastrointestinal disorders [87]. The maternal infection and stress during pregnancy are two important causal agents of depression in the adolescence of the descendants. Prenatal infection and stress are likely to make adolescents more susceptible to later insult, as stress, creating a latent vulnerability to depressive symptoms [88].

In general, we reported that MDD adolescents have higher levels of proinflammatory and anti-inflammatory cytokines than healthy volunteers, except for IL-10 and IL-1Ra. Up to now, the mechanism by which fluoxetine affects cytokine levels remains unclear. However, a temporary effect or fluctuations in cytokine levels has been observed during clinical follow-up in previous works [15, 34], such as the one reported here.

Week eight has been considered by several groups as a suitable time to evaluate the therapeutic efficacy of fluoxetine in MDD adolescents given the possibility of observing an improvement in the symptomatology at this time [89, 90]. However, we did not find a correlation between cytokine levels and the HDRS clinical score, which suggests that other physiological and psychological factors might contribute to the maintenance of the inflammatory tone in depressed patients. This should be considered in therapeutic management to improve the patient's quality of life.

It should be noted that some discrepancies in our results with respect to other reports may make sense if we consider the heterogeneity of sample sources, demographic data, sampling times, and diversity in analytical procedures used in each study.

Despite that our cytokine determinations were performed at systemic level, it is well known that chronic elevations in systemic cytokine levels impairs the permeability of the blood-brain barrier, facilitating the entry of cytokines into the brain [38, 39, 91, 92]. This issue is very important in the context of our work because, except for two anti-inflammatory cytokines: IL-10 and IL-1Ra, we found that both proinflammatory and anti-inflammatory cytokines were significantly elevated in MDD adolescents. This strongly suggests the possibility of altered cytokine effects not only at systemic inflammation level but also at the level of the central nervous system, which in turn may be closely related to the onset and development of major depressive disorder.

It is important to consider that some of the patients initially diagnosed with first-episode depression may later exhibit bipolar disorder. Therefore, we consider that an 8-week follow-up is a limitation to evaluate the probability of a change of diagnosis in this population (conversion to activation syndrome or bipolar disorder); however, none of our patients presented symptoms of activation syndrome or bipolar disorder during the treatment period. Although it has been reported that this conversion can occur at any time during SSRI treatment, there seems to be a tendency for it to appear during the first 2 or 3 weeks after drug initiation [93].

## 5. Conclusions

In this work, we found that MDD adolescents have higher levels of proinflammatory and anti-inflammatory cytokines than the healthy volunteers, except for IL-10 and IL-1Ra. To the best of our knowledge, this is the first report in adolescents with MDD in which alterations of IL-12, IL-13, and IL-15 are reported. Moreover, we described that fluoxetine treatment affects proinflammatory and anti-inflammatory cytokines in a temporal manner; however, no correlation was detected between the cytokine levels and the HDRS clinical score. The background of the relationship between cytokines and depression suggests that the altered inflammatory profiles that we observed in MDD adolescents may be contributing to the physiopathology of this disorder, although these inflammatory molecules are only one of the multiple biological alterations present in this disease.

## 6. Limitations of the Study

The group of healthy volunteers had to include subjects of legal age (age 18+), according to Mexican law; otherwise, recruiting healthy underaged adolescents willing to take part in the study would have been complicated since the authorization of parents or guardian is required. In fact, recruiting underaged adolescents with MDD was complicated since the authorization of parents or guardian is required; thus, the sample size obtained was small.

## Figures and Tables

**Table 1:** Demographic data.

	Healthy volunteers; *n* = 18	MDD adolescents; *n* = 22
Age (years)	18.9 ± 1.2	17.1 ± 2.3
Gender (male/female)	4/14	4/18
BMI (kg/m^2^)	23.2 ± 2.1	23.1 ± 2.1
Education (years)	12.9 ± 1.2	11.5 ± 2.6
Family history (yes/no)	3/15	8/14
First episode	NA	8
Recurrent episode	NA	14

Values are presented as mean ± standard deviation. Statistical analysis was performed by Student's *T*-test.

## Data Availability

The cytokine levels and psychiatric values used to support the findings of this study are available from the corresponding author upon request.
